# Survival Analysis and Risk Factors in COVID-19 Patients

**DOI:** 10.1017/dmp.2021.82

**Published:** 2021-03-25

**Authors:** Wen Lu, Shuhui Yu, Hailing Liu, Lihua Suo, Kuanyin Tang, Jitao Hu, Yantong Shi, Ke Hu

**Affiliations:** 1 Department of General Practice, People’s Hospital of Rizhao, Affiliated Clinical Hospital of Jining Medical University, Jining Medical University, Rizhao, Shandong, China; 2 Department of Critical Care Medicine, Renmin Hospital of Wuhan University, Wuhan, China; 3 Department of Respiratory and Critical Care Medicine, Renmin Hospital of Wuhan University, Wuhan, China; 4 Endocrinology Department, People’s Hospital of Rizhao, Affiliated Clinical Hospital of Jining Medical University, Jining Medical University, Rizhao, Shandong, China; 5 Department of Infectious Disease, People’s Hospital of Rizhao, Affiliated Clinical Hospital of Jining Medical University, Jining Medical University, Rizhao, Shandong, China; 6 Department of Neurology, Zhongnan Hospital, Wuhan University, Wuhan, China; 7 Department of Respiratory and Critical Care Medicine, People’s Hospital of Rizhao, Affiliated Clinical Hospital of Jining Medical University, Jining Medical University, Rizhao, Shandong, China

**Keywords:** COVID-19, risk factors of death, survival probability

## Abstract

**Objective:**

The aim of this study is to evaluate the clinical characteristics and outcomes in 2019 coronavirus disease (COVID-19) patients and to help clinicians perform correct treatment and evaluate prognosis and guide the treatment.

**Methods:**

Patients totaling 239 were diagnosed with COVID-19 and were included in this study. Patients were divided into the *improvement* group and the *death* group according to their outcome (improvement or death). Clinical characteristics and laboratory parameters were collected from medical records. Continuous variables were tested by an independent sample T test, and categorical variables were analyzed by the chi-square test or Fisher’s exact test. The Cox proportional hazard regression model was used for survival analysis in *death* patients. The time-dependent area under curves (AUC) based on white blood cell count, lymphocyte count, neutrophil count by age, blood urea nitrogen, and C-reactive protein were plotted.

**Results:**

Efficacy evaluation indicated that 99 (41.4%) patients had deteriorated, and 140 (58.6%) patients had improved. Oxygen saturation, hemoglobin levels, infection-related indicators, lymphocyte and platelet counts, C-reactive protein, serum albumin, liver and kidney function, and lactate dehydrogenase in improvement group were statistically significant between the *improvement* and *death* groups. A survival analysis revealed that comorbidities, lymphocyte counts, platelet count, serum albumin, C-reactive protein level, and renal dysfunction may be risk factors in patients with COVID-19.

**Conclusion:**

Patients with comorbidities, lower lymphocyte counts in hemogram, platelet count and serum albumin, high C-reactive protein level, and renal dysfunction may have higher risk for death. More attention should be given to risk management in the progression of COVID-19.

## Introduction

Coronavirus disease (COVID-19) broke out in 2019, and the virus that causes it, severe acute respiratory syndrome coronavirus 2 (SARS-CoV-2), spread rapidly. Confirmed cases could be found in countries around the world. The novel coronavirus has a raised spike glycoprotein on its surface. The S glycoprotein of the virus binds to angiotensin-converting enzyme 2 of the recipient cell, which causes the virus to enter the cell. Viruses can also enter cells through membrane fusion and release nucleic acids to synthesize new viruses, causing cytopathic disease and death, thus leading to tissue and organ lesions. This novel coronavirus is mainly related to pulmonary infection, which can involve multiple organs, such as liver, spleen, cardiovascular and cerebrovascular systems, stomach, and esophagus, and so on. In serious cases, symptoms can develop into acute respiratory distress syndrome, metabolic acidosis and multi-organ dysfunction syndrome, or even death. The initial clinical symptoms are mostly fever, dry cough, and fatigue. A few patients are accompanied by nasal congestion, runny nose, pharyngeal pain, myalgia, and diarrhea, and some patients may have no obvious symptoms. Previous studies suggest that the most prevalent comorbidities were hypertension (17%), diabetes mellitus (8%), and cardiovascular disease (5%) in COVID-19.^1^ It is important to evaluate patients’ clinical symptoms based on current influencing factors and comorbidities. Therefore, it is necessary to evaluate possible factors that may result in the progression or improvement of COVID-19 patients. Therefore, the influencing factors of status for COVID-19 patients and the result were investigated and analyzed in order to provide guidance about the treatment of this disease.

## Patients and Methods

The requirement for written informed consent was waived in consideration of emerging infectious diseases. The study included data from 239 COVID-19 patients in a hospital in Renmin Hospital of Wuhan University in China from December 31, 2020, to April 20, 2020.

### Diagnostic Criteria

Real-time fluorescent RT-PCR was used to detect SARS-CoV-2 in nasal or pharyngeal swab specimens, and patients with positive results twice were included in the study. In addition, relevant clinical symptoms, laboratory parameters, and major treatments were also included. The outcomes of the patients included improvement to discharge criteria or worsening to death. The patient discharge criteria and clinical type were based on COVID-19 diagnosis and treatment protocol version 7.^2^ Clinical types included (1) mild cases in which the patient had mild clinical symptoms and no imaging findings of pneumonia; (2) common cases in which the patient had fever, respiratory symptoms, and imaging manifestations of pneumonia; (3) severe cases in which the patient met any of the following criteria: (a) shortness of breath, RR ≥ 30/min; (b) fingertip oxygen saturation ≤ 93% at rest; (c) arterial partial pressure of oxygen (PaO2)/oxygen concentration (FiO2) ≤ 300 mmHg (1 mmHg = 0.133 kPa; areas with high altitudes, over 1000 m, were corrected for PaO2/FiO2 according to the formula: PaO2/FiO2 [atmospheric pressure (mmHg)/760]); (d) pulmonary imaging showing significant progression of > 50% within 24–48 hours; and (4) critical cases demonstrating one of the following conditions: (a) the patient developing respiratory failure and requiring mechanical ventilation; (b) shock; and (c) complication with other organ failure, requiring monitoring and treatment in the intensive care unit.

### Clinical Characteristics and Therapeutic Modalities

The study recorded the following clinical symptoms: (1) upper respiratory tract symptoms, such as cough, runny nose, or sneezing; (2) oppression in chest or dyspnea; (3) muscle pain; (4) headache; (5) gastrointestinal symptoms, such as nausea, vomiting, and diarrhea; (6) comorbidities; (7) body temperature; (8) time from onset to admission; and (9) total time in hospital. Comorbidities included a history of pulmonary comorbidities (eg, chronic obstructive pulmonary disease [COPD], lung cancer, bacterial pneumonia, pulmonary heart disease), hypertension, coronary artery disease, diabetes, and cerebral infarction. Clinical characteristics included clinical symptoms, time from symptom onset to hospitalization, total length of hospitalization, treatment and medication, and disease outcome.

### Laboratory Parameters

Routine tests were performed to determine blood counts, liver and kidney function, creatinine, albumin, procalcitonin, C-reactive protein, and potassium levels. Oxygen saturation was monitored.

### Statistical Analysis

Statistical analysis was performed using SPSS.19 (IBM Corp, Armonk, NY) and R software (R Core Team, Vienna, Austria). The measurement data were statistically described by mean ± standard deviation and performed by the t test. Count data were described by frequency, and the chi-square test or Fisher’s exact test was used for the comparison between groups. Multivariable analyses to identify factors associated with death from COVID-19 patients were performed by the Cox proportional hazards regression model. *P* < 0.05 was considered statistically significant. Time-dependent area under curves (AUC) were plotted.

## Results

### Demographic Characteristics and Laboratory Parameters

The cohort consisted of 239 patients: 140 (58.6%) patients met discharge criteria and 99 (41.4%) patients eventually died. Among the patients, 122 (51%) were male and 117 (49%) were female. The median age was 57 (39, 71) years; 107 (44.8%) patients were ≥ 60 years of age. Patients were divided into the *improvement* group and *death* group according to their final outcomes. The majority of patients had low and moderate fever, whereas the remaining patients had high or no fever. A higher proportion of patients in the improvement group came to the hospital within 6 days of the onset of symptoms, as opposed to patients in the death group, who took longer than 6 days from symptom onset to treatment. The mean age of patients in the death group was significantly higher than that in the improvement group (70.00 ± 13.53 vs 47.47 ± 16.83, *P* < 0.001). Patients in the death group had higher rates of upper respiratory symptoms(coughing, runny nose, and sneezing), dyspnea, headache, muscle pain, gastrointestinal symptoms (vomiting and diarrhea), and comorbidities. There were statistically significant differences in hypertension, diabetes, and coronary heart disease among the comorbidities. Among clinical symptoms, there were statistically significant differences in upper respiratory tract symptoms and dyspnea. The proportion of males in the death group was higher than that in the improvement group, and the difference was statistically significant. All data of patients with demographic characteristics, clinical symptoms, and comorbidities were summarized in Table 1.

**Table 1.** Clinical characteristics and comorbidities in 2 groups of COVID-19 patients

Demographic and Clinical Characteristics	n(%)			*P*
	Improvement	Death	Total	
	(n = 140)	(n = 99)	(n = 239)	
Age(Years)				< 0.001
< 60 years	106(75.7%)	26(26.3%)	132(55.2%)	
> 60 years	34(24.3%)	73(73.7%)	107(44.8%)	
Sex				0.035
Male	63(45%)	59(59.6%)	122(51.0%)	
Female	77(55%)	40(40.4%)	117(49.0%)	
URS				< 0.001
Yes	77(55%)	86(86.9%)	163(68.2%)	
No	63(45%)	13(13.1%)	76(31.8%)	
Dyspnea				< 0.001
Yes	53(37.9%)	62(62.6%)	115(46.9%)	
No	87(62.1%)	37(37.4%)	124(51.9%)	
Muscle Pain				0.231
Yes	32(22.9%)	30(30.3%)	62(25.9%)	
No	108(77.1%)	69(69.7%)	177(74.1%)	
Headache				0.667
Yes	13(9.3%)	11(11.1%)	24(10.0%)	
No	127(90.7%)	88(88.9%)	215(90.0%)	
Gastrointestinal Symptoms				0.814
Yes	11(7.9%)	9(9.1%)	20(83.7%)	
No	129(92.1%)	90(90.9%)	219(91.6%)	
Hypertension				< 0.001
Yes	18(12.9%)	46(46.5%)	64(26.8%)	
No	122(87.1%)	53(53.5%)	175(73.2%)	
Diabetes				0.006
Yes	11(7.9%)	20(20.2%)	31(13.0%)	
No	129(92.1%)	79(79.8%)	208(87.0%)	
Coronary Heart Disease				< 0.001
Yes	4(2.9%)	17(17.2%)	21(8.8%)	
No	136(97.1%)	82(82.8%)	218(91.2%)	
Pulmonary Comorbidities				0.054
Yes	7(5%)	12(12.1%)	19(7.9%)	
No	133(95%)	87(87.9%)	220(92.1%)	
Cerebral Infarction				0.154
Yes	5(3.6%)	8(8.1%)	13(5.4%)	
No	135(96.4%)	91(91.9%)	226(94.6%)	
Temperature (°C)				0.076
< 37.3	31(22.1%)	13(13.1%)	44(18.4%)	
37.4-38	27(19.3%)	12(12.1%)	39(16.3%)	
38.1-39	69(49.3%)	64(64.6%)	133(55.6%)	
> 39	13(9.3%)	10(10.1%)	23(9.6%)	
Time to Admission (days)				< 0.001
> 6	61(43.6%)	68(68.7%)	129 (54.0%)	
≤ 6	79(56.4%)	31(31.3%)	110 (46.0%)	
Hospital Days (days)				< 0.001
> 7	117(83.6%)	26(26.3%)	143(59.8%)	
≤ 7	23(16.4%)	73(73.7%)	96(40.2%)	
Clinical Type				< 0.001
Light type	8(5.6%)	0(0.0%)	8(3.3%)	
Common type	127(91.4%)	6(6.1%)	133(55.6%)	
Severe type	9(6.5%)	8(8.1%)	17(7.1%)	
critical type	2(1.4%)	85(85.8%)	87(36.4%)	

*Notes*: Data described by frequency; URS = upper respiratory symptoms.

Laboratory indicators showed statistically significant differences between the death group and the improvement group, except for sodium and potassium. Patients in the death group had lower oxygen saturation, platelet count, serum albumin levels, higher white blood cell count, and higher lymphocyte count. AST and ALT, which represent liver functions, and blood urea nitrogen, which represents kidney functions, are higher in the death group, indicating that patients in the death group have organ dysfunction to a different extent. The laboratory parameters are shown in Table 2.

**Table 2.** Laboratory parameters in improvement group and fatally affected group

	n = 239(%)		*P*
	Improvement (n = 140)	Death (n = 99)	
SaO_2_(%)	92.43 ± 10.13	86.74 ± 10.09	< 0.001
Hemoglobin (g/L)	127.47 ± 21.36	121.05 ± 22.02	0.025
WBC(×10^9^/L)	5.16 ± 2.24	9.27 ± 5.79	< 0.001
Neutrophils (×10^9^/L)	3.89 ± 2.59	7.03 ± 5.41	< 0.001
Lymphocytes (×109/L)	1.08 ± 0.57	0.61 ± 0.38	< 0.001
Platelet count (×10^9^/L)	200.00 ± 70.85	165.37 ± 93.97	0.001
C-reactive protein (mg/L)	105.66 ± 63.88	21.39±20.36	< 0.001
Serum albumin (g/L)	41.47 ± 25.90	33.05±7.71	0.002
ALP (U/L)	60.88 ± 26.44	84.91±53.95	< 0.001
AST (U/L)	27.74 ± 17.07	55.19±39.96	0.001
ALT (U/L)	26.48 ± 24.64	38.73±34.04	< 0.001
Urea (mmol/L)	4.51 ± 1.90	9.03±5.37	< 0.001
Creatinine (g/L)	63.31 ± 33.72	134.56±258.12	0.007
LDH(U/L)	230.43 ± 105.00	491.21±199.05	< 0.001
Serum sodium (mmol/L)	136.96 ± 17.79	139.93±15.19	0.179
Serum potassium (mmol/L)	3.95 ± 0.40	4.13±1.07	0.112

*Notes*: Data presented as mean ± standard deviation; ALP = alkaline aminotransferase; ALT = alanine aminotransferase; AST = aspartate aminotransferase; BUN = blood urea nitrogen; LDH = lactate dehydrogenase; PCT = procalcitonin; WBC = white blood cell.

### Survival Analysis

**Figure 1.**
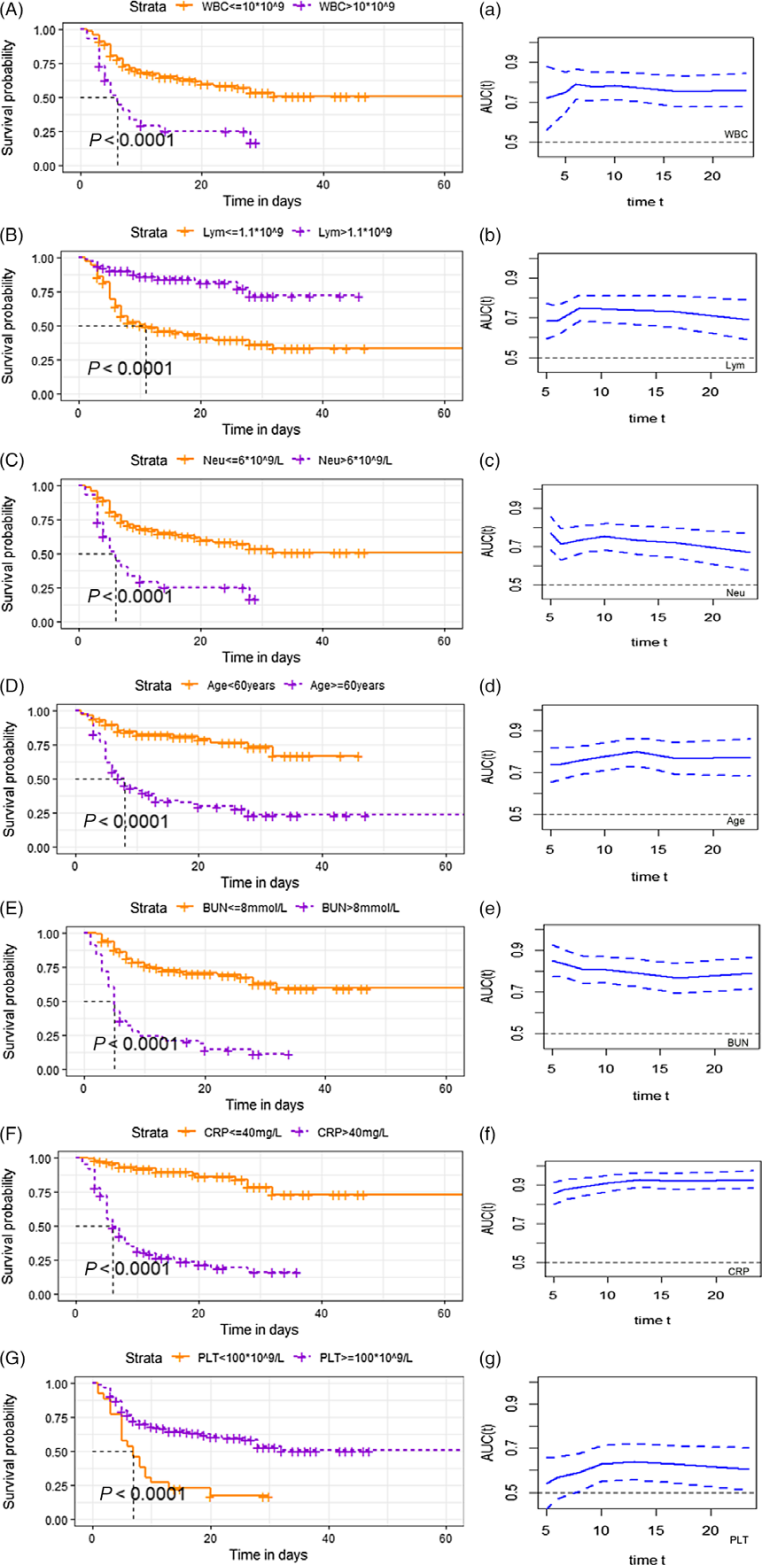
Survival curves probability based on risk factors. (A–G): Survival probability in patients with COVID-19 in different range of white blood cell (A), lymphocyte count (B), neutrophil counts (C), age (D), blood urea nitrogen (E), C-reactive protein (F) and platelet count (G). (a–g): Time dependent AUC of dead patients based on white blood cell (a), lymphocyte count (b), neutrophil counts (c), age (d), blood urea nitrogen (e), C-reactive protein (f) and platelet count (g). The x-axis starts from 20% to 80% of the total days. The value of AUC fluctuated with survival time. Dotted line represents the 95 percent confidence interval. Abbreviations: WBC, white blood cell; Neu, neutrophil counts; Lym, lymphocyte count; AST, alanineaminotransferase; ALB, serum albumin; BUN, blood urea nitrogen; Scr, serumcreatinine; CRP, C-reactive protein; PLT, platelet count.

Survival analysis showed that the risk of death was significantly increased in patients with upper respiratory symptoms, headache, hypertension, pulmonary comorbidities, lymphocyte count less than 1.1, platelet count lower than 100, C-reactive protein greater than 40 mg/L, serum albumin lower than 35g/L, and serum urea nitrogen more than 8 mmol/L. The survival curves based on white blood cell count, neutrophils, lymphocyte count, age, blood urea nitrogen, and C-reactive protein and platelet count are shown in Figure 1, A–F. Meanwhile, the time-dependent AUC curves based on these factors were plotted in Figure1a-f. The value of AUC is greater than 0.5, which is of good predictive significance.

## Discussion

SARS-CoV-2 is the seventh known human coronavirus.^3^ The new virus has an envelope, consisting of a single strand of RNA.^4^ SARS-CoV-2 is thought to have originated in bats, with 89% to 96% of its nucleic acid sequence identical to that of the virus carried by bats.^5^ SARS-CoV-2 is mainly transmitted through droplets but can also be transmitted by aerosols.^6^ The median incubation period of the virus is 4–5 days, and 97.5% of patients develop symptoms at 11.5 days after infection. At the start of the pandemic, the median period from clinical symptoms onset to progression to death is about 14 days.^7^ Morbidity and mortality varied from time to time and region to region, for reasons that may be related to virus variation, in addition to regional differences.

For its infectiousness, the number of cases increases exponentially without control measures. The hyperinflammatory response triggered by SARS-CoV-2 is the principal cause of death in infected patients.^8^ The most common symptoms are fever and hacking cough, and some patients present with runny nose, sneezing; a small number of patients develop nausea, vomiting, or diarrhea. Part of the population can have no symptoms or tends to improve after the infection, and some people can appear with rapid progression, such as respiratory failure or even death.^9^ The mortality increased significantly in patients with cardiovascular diseases, diabetes, COPD, and hypertension.^10^ Fatality increases with the progression of the severity of disease. We analyzed some relevant indicators and outcomes of patients diagnosed with COVID-19 in a hospital in Wuhan, in order to provide guidance for the treatment and prognosis of patients with coronavirus pneumonia.

The study included 239 patients with confirmed COVID-19. All patients were assessed until disease outcome (improved or dead). The study showed that 140 patients were discharged with a better health condition and 99 patients died. There was a statistically significant difference in age between the 2 groups, suggesting that old age is a risk factor for the disease, which is usually accompanied by a variety of comorbidities, resulting in complicated conditions.

Data from the Chinese Center for Disease Control and Prevention showed that 80% of patients experienced mild disease or even non-symptom and 20% developed severe symptoms, such as respiratory failure and multiple organ failure. Previous observational studies reported that patients with old age and comorbidities were more likely to deteriorate. It was later found that previously healthy patients also developed severe hypoxemia and respiratory failure, which were caused by inflammatory cascades.^8,11^ Pathological examination showed that lesions involved multiple organs in severe patients.^12^ Studies have shown that patients with COVID-19 pneumonia exhibit coagulation abnormalities, most commonly elevated levels of fibrinogen and D-dimer, often with mild thrombocytopenia.^13,14^ Typically mild thrombocytopenia is detected in 5–41.7% of COVID-19 patients.^15-17^ A meta-analysis of 7613 COVID-19 patients revealed that patients with severe disease had a lower platelet count than those with non-severe disease. Additionally, the non-survivors had a much lower platelet count than the survivors.^18,19^ Platelets play a significant role in inflammatory signaling in infectious response. Platelets may combine thrombotic and immune recruitment functions, thereby prevent microbial invasion. By combining thrombotic and immune recruitment functions, platelets may help focus hemostasis and immune responses against potential infectious agents to prevent microbial invasion.^20^

Studies have shown that inflammatory markers, C-reactive protein and neutrophil-to-lymphocyte ratio, are risk factors in COVID-19 patients.^21-23^ Excessive inflammatory responses, including high levels of cytokines, lymphocytosis, and mononuclear macrophage infiltration, are considered important reasons for the rapid progression of the disease.^8^ Our study found significant differences in white blood cell counts, neutrophil counts, and lymphocyte counts between the improvement group and the death group. C-reactive protein was also associated with the risk of death, suggesting that excessive inflammation is a risk factor for patients and should be controlled clinically. Given the consideration of the importance of white blood cell and neutrophil counts for COVID-19, we also plot the 2 factors in the survival curve.

In our study, survival analysis suggests that renal function factor, such as blood urea nitrogen, was associated with a risk of death in patients. The differences of urea nitrogen and creatinine between the improvement group and the death group were statistically significant. Data from complete autopsy, including histopathologic and virologic analysis in 12 patients who died from COVID-19, showed that high viral RNA titers were detected in the kidney.^24^ A study involving 701 patients with COVID-19 found that 5.1% patients experienced acute kidney injury.^25^ The mechanism involved acute tubular injury and may be associated with infiltration of lymphocytes and monocytes in renal tissue. In addition, cytokine injury and organ interference may also play a role in some biological processes. Patients with renal dysfunction who are infected with SARS-CoV-2 will face an increased risk of death, suggesting that effective renal function control should be a focus and addressed in the clinical treatment. Serum albumin levels partially reflect the body’s immune state. Patients with a bad nutritional status are more likely to experience lower serum albumin levels, which are harmful to the antibody production and virus clearance, and should be given high attention. One recent study suggested that poor nutritional status is a significant risk factor for severe COVID-19 infection.^11^ Severe anorexia and malnutrition may increase risks for respiratory failure and even require noninvasive ventilation.^26^

## Conclusion

The COVID-19 epidemic continues, and the challenges we face are still daunting. Assessment of the risk factors of the disease is helpful for clinicians to understand the risk of disease progression in a timely fashion, so as to carry out appropriate and multifaceted intervention earlier to achieve the best therapeutic purpose. Meanwhile, mass vaccination and the building of an immune barrier will be effective in combating the epidemic. There were a few limitations in our study. Our data sources were single-center and the sample size was not large enough to represent all infected people. Highly subjective outcomes such as pain may bias the assessment results. Patients without access to treatment were not included. Some patients were complicated with uremia, resulting in a large degree of dispersion of creatinine values in samples.
